# An Organic/Inorganic Nanomaterial and Nanocrystal Quantum Dots-Based Multi-Level Resistive Memory Device

**DOI:** 10.3390/nano11113004

**Published:** 2021-11-09

**Authors:** Sae-Wan Kim, JinBeom Kwon, Jae-Sung Lee, Byoung-Ho Kang, Sang-Won Lee, Dong Geon Jung, Jun-Yeop Lee, Maeum Han, Ok-Geun Kim, Gopalan Saianand, Daewoong Jung

**Affiliations:** 1 Advanced Mechatronics R&D Group, Korea Institute of Industrial Technology (KITECH), Daegu 42994, Korea; kei95304@gmail.com (S.-W.K.); jinbum0301@kitech.re.kr (J.K.); jdg8609@kitech.re.kr (D.G.J.); leejy@kitech.re.kr (J.-Y.L.); 2 Advanced Semiconductor Research Center, Gumi Electronics and Information Technology Research Institute (GERI), Gumi 39253, Korea; jslee1245@geri.re.kr (J.-S.L.); bhkang@geri.re.kr (B.-H.K.); 3 Daegu Technopark Daegu Smart Manufacturing Innovation Center, 46-17, Seongseogongdan-ro, Dalseogu, Daegu 42716, Korea; swlee@ttp.org; 4 School of Electronics Engineering, College of IT Engineering, Kyungpook National University, 80, Daehak-ro, Buk-gu, Daegu 41566, Korea; mehan@knu.ac.kr (M.H.); ogkim6441@gmail.com (O.-G.K.); 5 Global Centre for Environmental Remediation (GCER), College of Engineering, Science and Environment, The University of Newcastle, Callaghan, NSW 2308, Australia; SaiAnand.Gopalan@newcastle.edu.au

**Keywords:** CdSe/ZnS quantum dots, multi-level memory, PEDOT:PSS, ZnO nanoparticles

## Abstract

A cadmium selenide/zinc sulfide (CdSe/ZnS) quantum dot (QD)-based multi-level memory device with the structure [ITO/PEDOT:PSS/QDs/ZnO/Al:Al_2_O_3_/QDs/Al] was fabricated via a spin-coating method used to deposit thin films. Two layers of QD thin films present in the device act as charge storage layers to form three distinct states. Zinc oxide (ZnO) and aluminum oxide (Al_2_O_3_) were added to prevent leakage. ZnO NPs provide orthogonality between the two QD layers, and a poly(3,4-ethylenedioxythio-phene): poly(styrenesulfonate) (PEDOT:PSS) thin film was formed for effective hole injection from the electrodes. The core/shell structure of the QDs provides the quantum well, which causes the trapping of injected charges. The resistance changes according to the charging and discharging of the QDs’ trap site and, as a result, the current through the device also changes. There are two quantum wells, two current changes, and three stable states. The role of each thin film was confirmed through I–V curve analysis and the fabrication conditions of each thin film were optimized. The synthesized QDs and ZnO nanoparticles were evaluated via X-ray diffraction, transmission electron microscopy, and absorbance and photoluminescence spectroscopy. The measured write voltages of the fabricated device were at 1.8 and 2.4 V, and the erase voltages were −4.05 and −4.6 V. The on/off ratio at 0.5 V was 2.2 × 10^3^. The proposed memory device showed retention characteristics of ≥100 h and maintained the initial write/erase voltage even after 200 iterative operations.

## 1. Introduction

As the number of connected devices per person exceeds one, the demand for data in the world increases rapidly. With the miniaturization of devices, there is a need for storage devices with a high degree of integration to effectively store data [1,2]. In addition, as devices possessed by individuals become flexible, demand for flexible storage devices steadily increases [3,4]. Therefore, static random-access memory (SRAM), dynamic RAM (DRAM), and flash memory, which are existing sources of mainstream memory, are characterized by high storage efficiency and advanced manufacturing technology, but their integration and flexibility are also secured [5,6]. As an alternative to solving such a problem, studies on next-generation memories are actively being conducted [7,8,9]. Next-generation memories include magnetic-RAM (MRAM), phase-change RAM (PRAM), Ferroelectric RAM (FeRAM), and resistiveRAM (RRAM), each of which has parts to be improved, but solves the aforementioned problems. Among them, RRAM has a simple structure of electrode/resistive layer/electrode, and it is easy to apply each layer as a flexible material; thus, it has been developed by many researchers [10]. RRAM uses oxidebased materials such as NiO, TiO_x_, CuO_x_, ZrO_x_, and HfO_x_ as a resistive layer. It is advantageous to provide flexibility, but there is a limit to miniaturization because a minimum size must be secured to operate as a resistor [11,12,13,14,15]. Accordingly, an RRAM in which nanoscale materials such as quantum dots (QDs) and organic/inorganic materials are mixed has been proposed as an alternative [16,17,18,19]. QDs are semiconductor materials with a size of 2 to 10 nm and consist of a core that determines the band gap of the QDs, a shell that protects the unstable core, and a ligand that determines the reactivity of the QDs [20,21]. In particular, the QDs includes a quantum well with a core/shell structure, and can thus be used as a substitute for an oxide-based resistive layer [22,23,24,25,26,27,28]. Depending on whether charges are trapped in the quantum well, the conductivity (resistance) of the charge storage layer (CSL) composed of QDs changes. Before the trap site is filled, a small current flows through the device as external charges are trapped in the QDs (high resistance state, HRS); however, after the trap site is filled, it is no longer trapped and a large current flows through the device (low resistance state, LRS) [22,29,30]. The charge trapped in the quantum well is due to a high energy barrier, so de-trapping does not occur until [3] an external voltage is applied. This difference in current can be applied to memory devices. A charge barrier can be formed outside the CSL in order to suppress the light emission characteristics that may occur in the QDs and to suppress leakage in the shell of the partially weak QDs [31,32,33,34]. In addition, through such isolation, a plurality of CSL (quantum wells) can be formed, which enables the formation of a plurality of states and the development of a memory device capable of expressing multiple levels. Since the isolation layer should be able to form a charge barrier, not just for isolation purposes, metal oxides such as ZnO and Al_2_O_3_ with appropriate energy levels are used [35,36,37,38,39,40]. Through this, QDs-based RRAM can have high flexibility compared to oxide-based devices, and when a charge storage layer is formed with a single QDs, the size and area of the device can be ultimately downsized to the size of the QDs. In this paper, we propose RRAM, a next-generation memory based on QDs. The fabricated device has a structure of [ITO/PEDOT:PSS/QDs/ZnO/Al:Al_2_O_3_/QDs/Al], and it is a memory device for the current difference that occurs as charges are constrained in a quantum well existing in a QDs. A memory device capable of expressing a three-level state (low/middle/high) with a storage density characteristic of 3 to the power of n (3^n^) in ternary system was fabricated by forming two charge-storage layers (quantum wells). The QDs and ZnO NPs used were synthesized directly, and X-ray diffraction (XRD), transmission electron microscopy (TEM), photo-luminescence (PL), and absorption characteristics were analyzed to evaluate the properties of the synthesized material. For each thin film, a solution process was used—a spin-coating method—and the IV characteristics of the device according to the addition of each thin film were analyzed to optimize the device fabrication conditions. In addition, it took 100 h to evaluate the life characteristics and durability of the proposed device. The retention characteristics and the operating voltage characteristics were analyzed according to 200 repetitions.

## 2. Experimental Section

### 2.1. Synthesis of Colloidal Quantum Dots (QDs)

**Figure 1:**
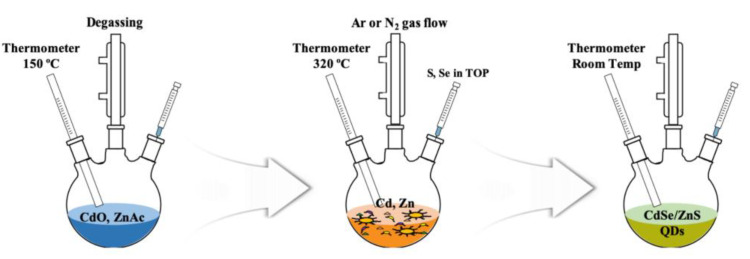
Schematic diagram of the synthesis of green light-emitting CdSe/ZnS quantum dots.

The CdSe/ZnS quantum dots (QDs) used as the charge storage layer were synthesized through the following process, which is schematically shown in Figure 1. The synthesized QDs have a core/shell structure; the core is composed of CdSe and the shell is composed of ZnS, and has a green emission wavelength [41,42]. First, 4 mmol of Zn(CH_3_COO)_2_·2H_2_O, 0.3 mmol of CdO, and 5 mL of OA were mixed in a three-neck flask, heated at 150 °C, with flowing Ar gas for 30 min. After raising the temperature to 320 °C, 2 mL of trioctylphosphine (TOP) with 0.2 mM Se and 3.5 mM S was rapidly injected. After that, it was maintained at 300 °C for about 10 min and the QDs were synthesized in a core/shell structure. In order to remove impurities and reactants generated during the synthesis, ethanol and toluene were injected into the solution in a 1:1 ratio and centrifuged at 3000 rpm. Through centrifugation, a solution containing impurities and pure QDs was separated, and pure QDs were obtained by removing the solution containing impurities and removing the moisture. The remaining QDs were weighed and finally dispersed in toluene at an appropriate concentration (20 mg/mL).

### 2.2. Synthesis of Semiconductor Nanocrystal ZnO Nanoparticles (NPs)

**Figure 2:**
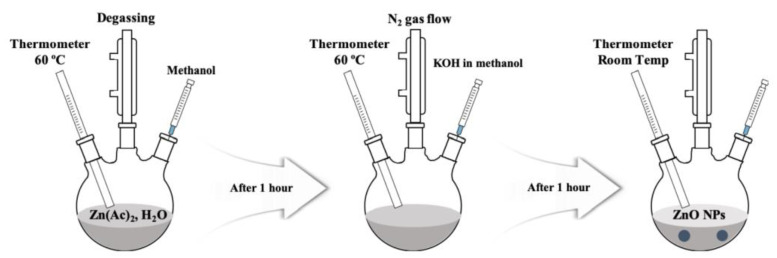
Schematic diagram of the synthesis of ZnO nanoparticles.

Using the sol–gel method, semiconductor ZnO nanoparticles (NPs) to be used as hole barriers were synthesized and the synthesis process is shown in Figure 2 [43,44]. In order to make precursors Zn^2+^ and OH^−^, zinc acetate dihydrate (Zn(CH_3_COO)_2_·2ּH_2_O, Sigma-Aldrich, St. Louis, MO, USA) and potassium hydroxide (KOH, Sigma-Aldrich, St. Louis, MO, USA) were dissolved in methanol at 60 °C, respectively. After dissolving by heating for 1 h, the methanol in which KOH was dissolved was slowly injected into the solution in which zinc acetate was dissolved. After 1 h of reaction, ZnO NPs with a size of 3 to 5 nm were synthesized. The next step was to perform a cleaning process to remove impurities generated during the synthesis process. Isopropanol (IPA) and hexane were injected at a ratio of 1:1:5 to remove K^+^ ions corresponding to by-products. After incubation at room temperature for 24 h, impurities and ZnO NPs were separated into two layers due to the difference in density, and pure ZnO NPs were obtained through centrifugation. Finally, the synthesized ZnO NPs were dispersed in ethanol at an appropriate concentration (20 mg/mL) [45].

### 2.3. Formation of QDs/Al Nanocluster Based Memory Device

**Figure 3:**
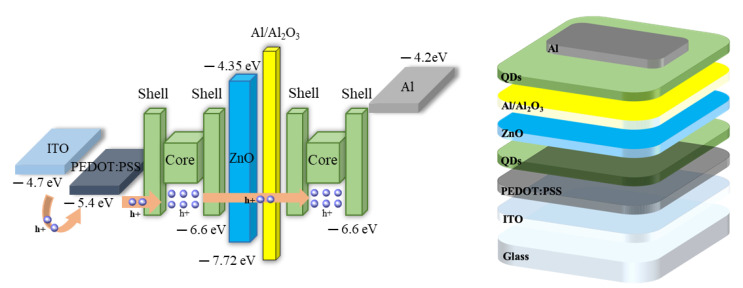
Schematic diagram and the energy band diagram of fabricated memory devices.

Indium tin oxide (ITO) patterned on glass was used as the lower electrode, and cleaning was performed in the order of acetone/methanol/IPA to remove impurities and PRs present on the surface. UV–Ozone treatment was performed for 15 min in order to improve the bonding strength and surface characteristics with the upper thin film [46]. Next, a PEDOT:PSS (Clevios AI 4083, Dayton, OH, USA) thin film with a work function of 5.4 eV was formed using a spin-coating method (3000 rpm, 30 s), and annealing was performed (150 °C, 10 min). PEDOT:PSS can reduce the energy gap between a QDs with a work function of 6.6 eV and an ITO with a work function of 4.7 eV, and can be used as a hole injection layer (HIL) because it has high mobility for holes [46]. The QDs thin film corresponding to the first CSL was formed by the same spin-coating method (1500 rpm, 30 s), and annealing (80 °C, 30 min) was performed in a vacuum oven. ZnO NPs thin film corresponding to the hole-blocking layer (HBL) was deposited on the QDs thin film by spin-coating method (1500 rpm, 30 s), and annealing (90 °C, 30 min) was performed in vacuum oven. Al with a thickness of 25 nm was deposited to form a nanocluster (NC) using thermal deposition, and was reacted in the air at room temperature for 3 h to form an aluminum oxide (Al_2_O_3_) layer [47]. Additionally, the QDs thin film corresponding to the second CSL was formed through the same method as the first QDs thin film. Finally, an aluminum layer (over 250 nm) corresponding to the upper electrode was deposited using thermal deposition. Figure 3 shows a schematic diagram of the device fabrication process and energy level of the fabricated device.

## 3. Results and Discussion

### 3.1. Optical and Physical Properties of the Synthesized QDs

**Figure 4:**
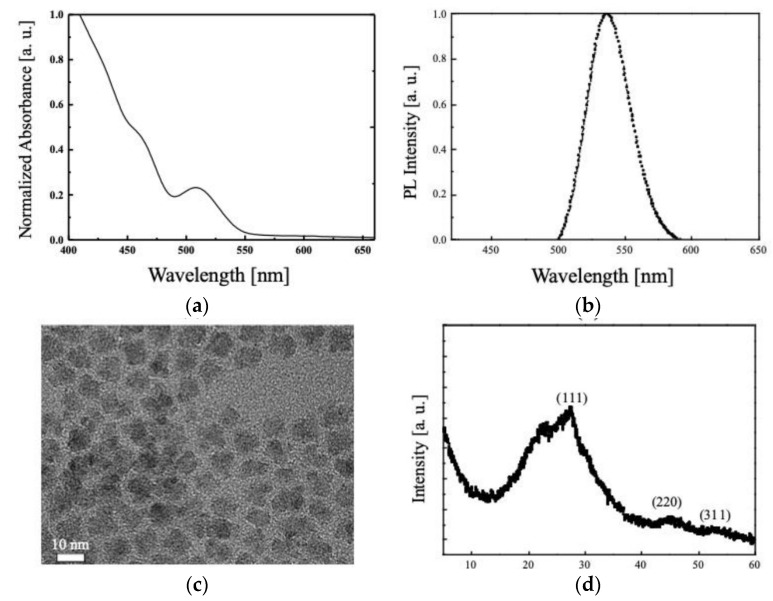
The measured results: (**a**) photoluminescence (PL) characteristics, (**b**) absorbance properties, (**c**) transmission electron microscopy (TEM) image, and (**d**) X-ray diffraction (XRD) spectrum of synthesized quantum dots (QDs).

Photoluminescence (PL) spectrum, absorbance, X-ray diffraction (XRD) of synthesized QDs, and transmission electron microscopy (TEM) images of the synthesized QDs thin films were taken to evaluate the synthesis success and characteristics of the synthesized QDs. The measured results are presented in Figure 4. As can be seen in Figure 4a, synthesized QDs have an absorption peak at 515 nm and high absorbance in the UV region. In the PL spectrum of Figure 4b, it can be seen that the peak at 536 nm. Additionally, from the TEM image shown in Figure 4c, it can be seen that the synthesized QDs have a size of about 6 nm. Through this, it was confirmed that QDs were successfully synthesized [43,48]. In addition, as can be seen in the measured XRD (Figure 4d) to confirm the crystal structure and crystallinity of synthesized QDs, the peak wavelengths corresponding to the (111), (220), and (311) planes are evident at 28°, 45°, and 50°, respectively. These results show that QDs formed a zinc blende structure. Additionally, the synthesized QDs have a core/shell structure, and charge can be trapped in the quantum well formed by this core/shell structure [27,49].

### 3.2. Properties of the Synthesized ZnO NPs

**Figure 5:**
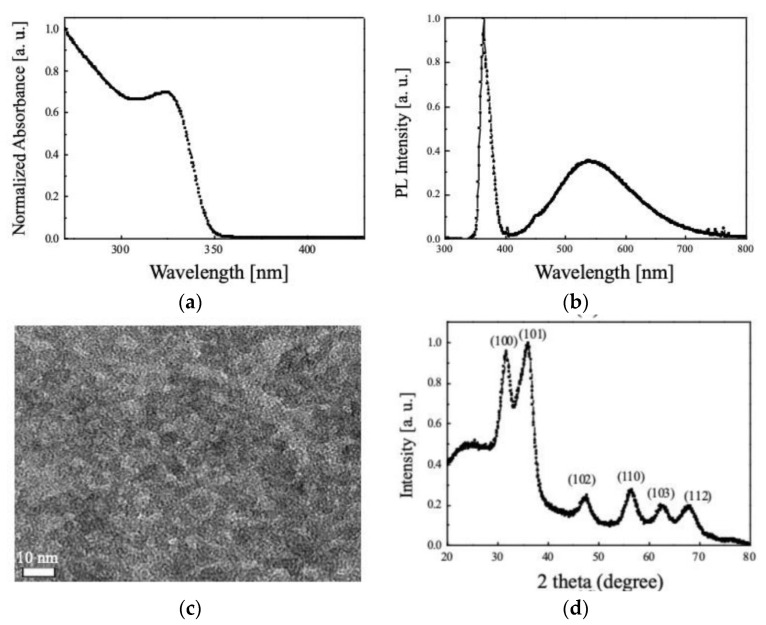
The measured results: (**a**) photoluminescence (PL) characteristics, (**b**) absorbance properties, (**c**) transmission electron microscopy (TEM) image, and (**d**) X-ray diffraction (XRD) spectrum of synthesized ZnO nanoparticles (NPs).

PL spectrum, absorbance, XRD of synthesized ZnO NPs, and TEM image of the synthesized ZnO NPs thin films were taken to evaluate the synthesis success and characteristics of the synthesized ZnO NPs. The measured results are presented in Figure 5. As can be seen in Figure 5a, synthesized ZnO NPs have an absorption peak at 323 nm and high absorbance in the UV region. In the PL spectrum of Figure 5b, it can be seen that the peak at 363 nm and broad characteristics appear in the visible region of 500 nm. Additionally, from the TEM image shown in Figure 5c, it can be seen that the synthesized ZnO NPs have a size of about 5 nm. Through this, it was confirmed that ZnO NPs were successfully synthesized [50]. In addition, as can be seen in the measured XRD (Figure 5d) to confirm the crystal structure and crystallinity of synthesized ZnO NPs, the peak wavelengths corresponding to the (100), (101), (102), (110), (103), and (112) planes are evident at 32°, 36°, 47°, 57°, 63°, and 67°, respectively. Through this, the synthesized ZnO NPs have a hexagonal crystal structure [51].

### 3.3. Nanocluster(NC) Verification

**Figure 6:**
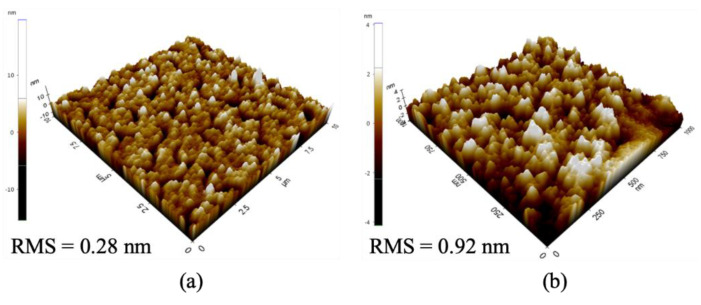
AFM image of. (**a**) QDs only, (**b**) QDs in the Al_2_O_3_ thin film.

The atomic force microscopy (AFM) image of the thin film was measured to confirm the nanocluster (NC) formed on the aluminum oxide layer and the QDs thin film, and the results are presented in Figure 6. As the QDs are embedded in the partially oxidized aluminum layer, NCs are formed. Since the formation of the NCs allows the QDs formed at the bottom and the QDs formed at the top to be isolated from each other, it can be applied as a bi-stable memory [22,45,47]. In the AFM result of forming only QDs as a thin film in (Figure 6a), the root-mean-squared (RMS) roughness was 0.28, but the RMS roughness of the QDs thin film deposited on partially oxidized aluminum (Figure 6b) was 0.92, showing a significant increase. Through these results confirming that the RMS roughness has increased significantly, it can be confirmed that the QDs are embedded in partially oxidized aluminum and are physically isolated from the lower QDs to form NCs [45].

### 3.4. I-V Properties of Fabricated Memory Devices

**Figure 7:**
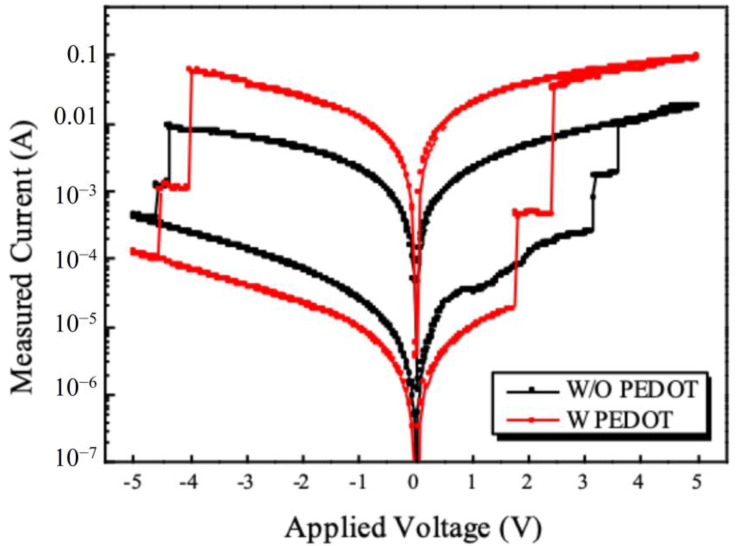
Current–voltage (I-V) characteristics of fabricated memory device.

In order to confirm the characteristics of the fabricated device, the current characteristics according to voltage were measured, and this is presented in Figure 7. As can be seen in Figure 7, when the voltage is swept from 0 to 5 V, it can be seen that three different current values appear, and each can be defined as a low/middle/high state. When the voltage increases from 0 to 5 V, holes are injected through the ITO electrode by the externally applied voltage and are confined to the core through the shell of the QDs. During this process, the trap sites present in the first QDs thin film are filled. When the first write voltage reaches 3.17 V, the trap sites present in the first QDs are all filled and no more traps occur. At this time, since the charge loss due to the trap disappears, the current flowing through the device, which decreases the resistance of the thin film, greatly increases, and this can be said to be transitioned from low to middle state. As the voltage continues to increase at the first write voltage, the second write voltage (3.6 V) is reached. In this process, the current value changes as charges are confined to the QDs existing in the second CSL. In this case, since no more charge trap occurs and the charge loss disappears, the resistance of the thin film decreases, resulting in an increase in current. Through this, a transition from middle state to high state occurs. After two transitions, a large current change does not appear even if the external voltage increases. This is because there is no more trap site, so it acts like a resistor and only a small amount of current change in the external voltage is observed. After that, even if the voltage is swept from 5 V to 0 V, the trap sites already in the two CSL are filled, and the high state is maintained because the positive voltage cannot de-trap these charges. At this time, it can be seen that the voltage to set to each middle/high state is 3.17 and 3.6 V, and the high/low ratio of the device identified at the read voltage (0.5 V) is 44. On the contrary, in the process of sweeping from 0 to −5 V, electrons are injected from the ITO by the negative voltage applied from the outside, and the injected electrons and trapped holes are recombined and the trapped holes disappear. In this process, as a void space is created in the trap site that was filled in, the resistance of the CSL increases significantly and the current flowing through the device decreases. Contrary to the above-described write process (voltage sweep at positive voltage), two rapid current changes appear in the CSL formed at two locations [11] by this principle. In this process, the first transition from the high state to the middle state and the second transition from the middle state to low state appear. These transitions appear at −4.4 and −4.6 V, respectively, and the charge trap does not occur in the process of returning from −5 to 0 V, so the low state is maintained. In order to improve the operating voltage and high/low ratio of the fabricated device, the I-V characteristics of the device to which the conductive polymer PEDOT:PSS is applied can be confirmed in Figure 7. PEDOT:PSS has high hole mobility, and a work function exists between the core of the QDs and ITO electrode, so that effective hole injection from the ITO electrode is possible. The shape of the I-V according to the positive/negative voltage sweep is the same as in Figure 7, but the write voltage of the device manufactured by applying PEDOT:PSS is greatly reduced to 1.8 and 2.4 V, respectively. In addition, due to the high hole mobility of PEDOT:PSS, the current corresponding to the high state increased significantly, and the overall high/low ratio was also significantly improved to 2200. However, in the case of the erase voltage, when PEDOT:PSS is applied, a slight change appears to −4.05/−4.6 V, respectively, but it can be confirmed that it does not decrease or increase significantly. It can be seen that PEDOT:PSS has high hole mobility and facilitates hole injection, but does not significantly affect the erase characteristics according to electron behavior because it does not significantly affect the mobility and injection of electrons. Through this, it can be seen that two trap sites are formed by the two CSL formed on the fabricated device, and accordingly, a memory device capable of expressing three states has been fabricated. In addition, by applying PEDOT:PSS, it was possible to significantly increase the operating voltage and high/low ratio of the fabricated device.

### 3.5. Retention Properties

**Figure 8:**
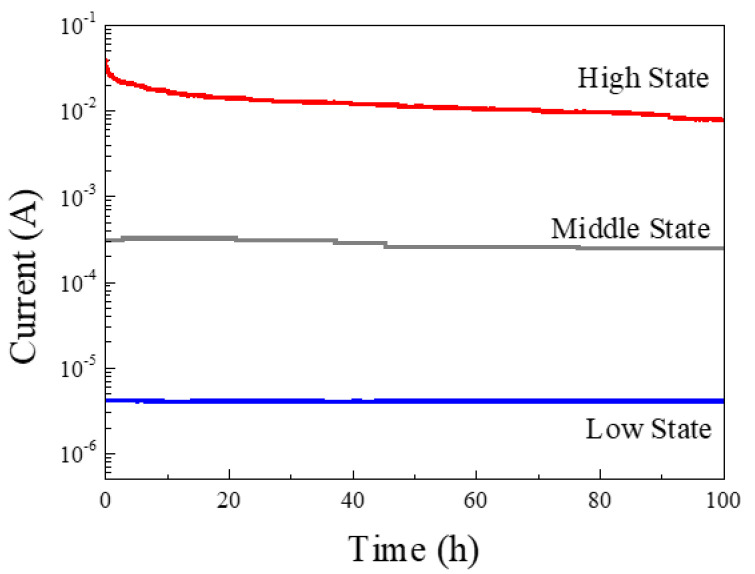
Retention characteristics of the fabricated memory device by measuring the current at 0.5 V after applying write voltage (2/2.7 V).

In order to check the lifespan characteristics of the fabricated memory device, the current of the device was measured over time, and the measured results are presented in Figure 8. The low state was measured immediately without the write process, and 2/2.7 V which is slightly higher than the write voltage, was applied so that the write could be performed; this was sufficient to set the middle/high state. To measure the current of each state, a read voltage (0.5 V) was applied. For 100 h, the high state showed a decrease in current by 0.03 A, the middle states indicated a decrease in the current by 0.585 × 10^−4^ A, and the low state indicated a decrease in current by 0.294 × 10^−4^ A. Additionally, it can be seen that, even after 100 h, the three states do not cross each other and maintain the initial current value. Through these results, it was confirmed that the fabricated device was a memory device capable of maintaining an initial state for 100 h.

### 3.6. Robustness Properties

**Figure 9:**
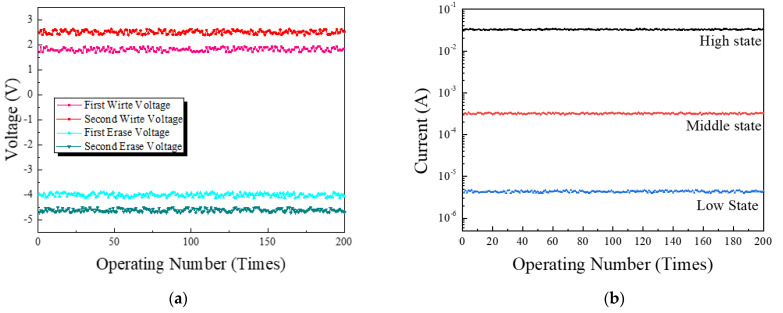
(**a**) Write/erase voltages, (**b**) state current of the fabricated memory device according to repeated operations.

In order to evaluate the robustness of the fabricated memory device, the operation voltage of the device was measured according to 200 repetitive write/erase processes. As shown in Figure 9a, a slight difference is observed depending on the repetitive operation, but this is a difference in the degree that can be seen as a measurement error, and it can be seen that all four operating voltages are maintained stably without significant change compared to the initial value. Additionally, in order to check the reliability of each state for repeated operation, the state current was measured by applying the write voltage and then applying the read voltage. As shown in Figure 9b, it was confirmed that, during 200 repetitions of operation, a difference of up to 4% was exhibited from the initial current value. Due to these results, it is confirmed that the fabricated device maintains the write/erase voltage and each state current even after 200 iterations, and it has high robustness.

## 4. Conclusions

In this study, a quantum dots (QDs)-based multi-level memory device was proposed in which a plurality of charge storage layers (CSL) was formed using QDs and aluminum oxide (Al_2_O_3_) film. Charges are confined to the quantum well existing in a QDs having a core/shell structure, and a current difference occurs according to the charge/discharge of these trap sites. It is a device capable of expressing three states through different current values expressed at this time. The QDs and zinc oxide nanoparticles (ZnO NPs) used in this study were synthesized directly, and the synthesis was confirmed by measuring photo-luminescence (PL), absorbance, transmission electron microscopy (TEM), and X-ray diffraction (XRD) of the synthesized material. Through the measurement results, it was confirmed that QDs and ZnO NPs were successfully synthesized. The fabricated device has the structure of [ITO/PEDOT:PSS/QDs/ZnO/Al:Al_2_O_3_/QDs/Al], and the upper and lower QDs are separated by ZnO NPs and Al_2_O_3_ thin films so that two charge storage layers can exist; as a result, it is a device capable of expressing three states. PEDOT:PSS formed at the bottom can reduce the energy gap between the electrode and the QDs to enable effective hole injection. The maximum on/off ratio of the manufactured device is 2.2 × 10^3^, the write voltage is 1.8/2.4 V, and the erase voltage is −4.05/−4.6 V. It was confirmed that the initial current value could be maintained without a large reduction, and it was confirmed that the initial operating voltage and state current were maintained even with 200 repeated operations. We propose a QDs-based memory device using an organic/inorganic mixed layer with high stability that can be applied as a next-generation memory device.
